# I Know I'm Healthy Because: Qualitative Definitions of Health in Older Rural African-Americans

**DOI:** 10.1093/geroni/igab046.3149

**Published:** 2021-12-17

**Authors:** Carolyn Adams-Price, Muhammed Riaz

**Affiliations:** 1 Mississippi State University, Mississippi State, Mississippi, United States; 2 Mississippi State University, Mississippi State University, Mississippi, United States

## Abstract

In recent years, there has been attention to health disparities between racial groups in the US, and between urban and residents. Older rural African Americans are at high risk, but have historical reasons tor distrust the health care system. This study examined qualitative definitions of health in older rural African Americans. Our sample included 47 African Americans aged 52-79 (20 male, 27 female, median age = 66 ) from non metropolitan counties in northeast Mississippi, at least 10 miles from the nearest town of more than 1000. Participants rated their health on a 5-point scale; only 1 person rated their health as a 5 for excellent. On average, they rated their health as fair. Most reported significant health problems, (mean=2) including 17 (36%) who reported having been diagnosed with diabetes. Participants were asked by interviewers “how they knew they were healthy.” Their responses were transcribed. Using phenomenological methods, participants’ responses were sorted into naturally-occurring categories, which were retested against the data. The categories that emerged were Performing Basic Tasks is Enough/I’m OK (12), Good Health Due to Healthy Behaviors (8), Healthy Due to Social Support or God (11), Healthy Despite One Problem (6), and I’m Not OK (7). Given that our sample is somewhat younger than most gerontological samples, participants seemed to have relatively low expectations about their health, which might not be surprising considering the health problems in the sample. Interventions to improve the health of this group should concentrate on increasing health self-efficacy and expectations.

